# Value of serial cervical length measurement in prediction of spontaneous preterm birth in post-conization pregnancy without short mid-trimester cervix

**DOI:** 10.1038/s41598-018-33537-1

**Published:** 2018-10-17

**Authors:** Liang Wang

**Affiliations:** Department of Ultrasound, Chinese Academy of Medical Sciences & Peking Union Medical College Hospital, 1 Shuaifuyuan Wangfujing, Beijing, 100730 China

## Abstract

Serial cervical length (CL) measurement in mid-trimester is recommended in post-conization pregnancy to estimate the risk of spontaneous preterm birth (SPTB). A short mid-trimester cervix (CL < 25 mm) has been considered as a strong predictor for SPTB. However, the low incidence of short cervix limits the utility of mid-trimester CL measurement in prediction of SPTB. A great proportion of women who develop SPTB don’t have a short mid-trimester cervix. Therefore, this study was aimed to investigate the additional value of serial CL measurement in predicting SPTB in addition to detecting short cervix alone. A total of 613 post-conization pregnant women who did not have short mid-trimester cervix between January 2004 and January 2014 were included in this study. Serial CL measurements were taken by transvaginal ultrasound at three timepoints (A: 13 + 0–15 + 6 weeks, B: 16 + 0–18 + 6 weeks, and C: 20 + 0–22 + 6 weeks). Eight parameters were analyzed for predicting SPTB, including CL measurements at different timepoints (CL_A_, CL_B_, CL_C_), the maximum and minimum CL measurements (CL_MAX_, CL_MIN_), and the percentage change in CL measurement between different timepoints (%ΔCL_AB_, %ΔCL_BC_, %ΔCL_AC_). After univariate and multivariate analysis, CL_MAX_ and %ΔCL_AC_ were independent variables in predicting SPTB. Lower CL_MAX_ (OR [95%CI]: 0.92 [0.90–0.93]) and higher %ΔCL_AC_ (OR [95%CI]: 1.05 [1.01–1.09]) were related to an increasing risk of SPTB. In conclusion, our study for the first time in literature reported the value of serial CL measurement in prediction of SPTB in post-conization pregnancy without short mid-trimester cervix. In the subpopulation of pregnant women who did not have short mid-trimester cervix, CL_MAX_ and %ΔCL_AC_ were of value in predicting SPTB, which warranted further investigations.

## Introduction

Conization is a cone biopsy of the uterine cervix that serve as a diagnostic and therapeutic procedure, which is used for conservative treatment of cervical intraepithelial neoplasia (CIN)^1^. However, this local cervical treatment has been correlated to an increased risk of spontaneous preterm birth (SPTB) in a subsequent pregnancy^2^. The underlying pathogenesis is poorly understood. Hypotheses include a loss of mechanical support from the cervix, a change in immunologic defense mechanism, and an alteration in cervicovaginal bacterial flora^2–4^.

Currently, transvaginal cervical sonography is commonly utilized in the mid-trimester to assess CL in women at high risk for SPTB, such as post-conization pregnacy^5^. Detection of a short cervix has been considered to be a surrogate for cervical insufficiency, and therefore, a useful predictor of SPTB^3,6^. In clinical practice, once a woman has been identified as being at higher risk of SPTB due to short cervix, a decision regarding prophylactic treatment must be made^7^. A cut-off value of 25 mm is widely used as the threshold^8,9^.

However, the low incidence of short cervix limits the utility of mid-trimester cervical screening in prediction of SPTB. A great proportion of women who developed SPTB did not have a short mid-trimester cervix^10^. For example, Boelig *et al*. reported the majority of women (82%) who developed SPTB did not have a short mid-trimester cervix in a prospective cohort study, which included 2071 singleton pregnant women without a prior history of SPTB undergoing universal CL screening in mid-trimester^11^.

In fact, little was known about the additional value of mid-trimester cervical screening in prediction of SPTB in addition to detecting short cervix alone. In the subpopulation of pregnant women who did not have short mid-trimester cervix, the predictability of SPTB was of great clinical value, however only limited studies focused on this issue in previous literatures. Therefore, the present study was designed to investigate the value of serial CL measurement in prediction of SPTB in post-conization pregnancy without short mid-trimester cervix.

## Results

A total of 613 pregnant women post-conization were included in this retrospective study. The average maternal age was 34.2 ± 5.1 years. Ethnicity information was available in 578 women (94.2%). Of them, 145 (25.1%) were Asian, 117 (20.2%) were Black, and 316 (54.7%) were Caucasian. For pregnancy outcome, despite the absence of short cervix <25 mm in mid-trimester, SPTB < 37 weeks still occurred in 45 women (7.3%).

### Predictor selection

A total of eight parameters were taken as the candidates for SPTB predictors, including CL measurements at different timepoints (CL_A_, CL_B_, CL_C_), the maximum and minimum CL measurements (CL_MAX_, CL_MIN_), and the percentage change in CL measurement between different timepoints (%ΔCL_AB_, %ΔCL_BC_, %ΔCL_AC_). In univariate analysis, CL_C_, CL_MAX_, CL_MIN_, and %CL_AC_ were significantly different between SPTB group and term birth group (Table 1). In multivariate analysis, CL_C_ and CL_MIN_ were excluded on logistic regression, while CL_MAX_ and %CL_AC_ were finally selected as the predictors for SPTB (Table 1).

**Table 1:** Predictors selection in univariate and multivariate analysis.

	Univariate analysis			Multivariate analysis	
	SPTB < 37 weeks (n = 45)	Term birth ≥37 weeks (n = 568)	P value	OR (95%CI)	P value
CL_A_	33.0 ± 4.9 mm	34.2 ± 4.4 mm	0.072	—	—
CL_B_	32.0 ± 4.7 mm	33.1 ± 4.5 mm	0.106	—	—
CL_C_	30.6 ± 3.9 mm	32.0 ± 4.1 mm	0.033	1.08 (0.75–1.55)	0.675
CL_MAX_	33.2 ± 4.9 mm	34.9 ± 4.5 mm	0.013	0.92 (0.90–0.93)	0.000
CL_MIN_	30.3 ± 3.9 mm	31.7 ± 4.1 mm	0.020	0.95 (0.62–1.45)	0.793
%ΔCL_AB_	4.1 ± 6.9%	3.0 ± 7.3%	0.396	—	—
%ΔCL_BC_	3.7 ± 7.0%	2.2 ± 8.0%	0.234	—	—
%ΔCL_AC_	8.5 ± 7.0%	5.2 ± 9.7%	0.048	1.05(1.01–1.09)	0.024

SPTB: spontaneous preterm birth; CL: cervical length; CI: confidence interval; OR: odds ratio; CL_A_/CL_B_/CL_C_:CL measurements at different timepoints; CL_MAX_/CL_MIN_: maximum and minimum CL measurements; %ΔCL_AB_/%ΔCL_BC_/%ΔCL_AC_: percentage change in CL measurement between different timepoints; Screening timepoints: A 13 + 0~15 + 6 weeks, B 16 + 0~18 + 6 weeks, C 20 + 0~22 + 6 weeks.

### Diagnostic performance

Using CL_MAX_ and %ΔCL_AC_ as the predictors of SPTB, their area under curve (AUC) of receiver operating characteristic (ROC) curve were 0.611 and 0.600, respectively. The sensitivity, specificity, positive predictive value (PPV), negative predictive value (NPV) and likelihood ratios (LR) of CL_MAX_ and %ΔCL_AC_ in predicting SPTB were shown in Table 2 with different cut-off values. According to the ROC curve, the cut-off values with the optimum balance between sensitivity and specificity were ≥10% for %ΔCL_AC_ (Sensitivity = 42.9%, Specificity = 71.2%, PPV = 9.7%, NPV = 94.5% and LR = 1.49) and <30 mm for CL_MAX_ (Sensitivity = 26.7%, Specificity = 90.8%, PPV = 18.8%, NPV = 94.0% and LR = 2.91), respectively.

**Table 2:** Sensitivity, specificity, positive predictive value, negative predictive value and likelihood ratio of %ΔCL_AC_ and CL_MAX_ in predicting SPTB.

		Sensitivity	Specificity	PPV	NPV	LR (95%CI)
%ΔCL_AC_ (n = 518)	≥0%	94.3%	20.1%	7.9%	98.0%	1.18 (1.09–1.28)
	≥5%	65.7%	44.7%	7.9%	94.7%	1.19 (0.99–1.42)
	≥10%	42.9%	71.2%	9.7%	94.5%	1.49 (1.18–1.88)
	≥15%	22.9%	82.4%	8.6%	93.6%	1.30 (0.99–1.70)
CL_MAX_ (n = 613)	<30 mm	26.7%	90.8%	18.8%	94.0%	2.91 (2.24–3.79)
	<35 mm	57.8%	50.4%	8.4%	93.8%	1.16 (0.95–1.42)
	<40 mm	91.1%	13.6%	7.7%	95.1%	1.05 (0.96–1.16)
	<45 mm	97.8%	3.0%	7.4%	94.4%	1.01 (0.96–1.06)

SPTB: spontaneous preterm birth; CL: cervical length; PPV = positive predictive value; NPV = negative predictive value; LR = likelihood ratio; CI: confidence interval; CL_MAX_: maximum CL measurement; %ΔCL_AC_: percentage change in CL change between timepoint A and C; Screening timepoints: A 13 + 0~15 + 6 weeks, B 16 + 0~18 + 6 weeks, C 20 + 0~22 + 6.

## Discussion

The value of mid-trimester cervical screening in predicting the risk of SPTB has been widely studied. A mid-trimester CL < 25 mm is considered “short,” as 25 mm corresponds to the 10th percentile for this gestational age^12^. A short mid-trimester cervix has been considered as one of the strongest risk factors for SPTB^10^.

However, the low incidence of short cervix limits the utility of mid-trimester CL measurement in prediction of SPTB. Esplin *et al*. reported a multicenter, prospective observational cohort study that included 9410 nulliparous singleton pregnancies. Of them, 474 (5.0%) had SPTB, 335 (3.6%) had medically indicated preterm births, and 8601 (91.4%) had term births. Among women with SPTB < 37 weeks, short CL < 25 mm occurred in only 23.3% cases in the mid-trimester^13^. Furthermore, even in high-risk population, a great proportion (31–81%) of women who developed SPTB did not have a short CL < 25 mm during mid-trimester cervical screening^12^.

Unfortunately, in addition to detecting short cervix, little was known about the additional value of mid-trimester cervical screening in predicting SPTB. In the subpopulation of pregnant women who did not have short mid-trimester cervix, the predictability of SPTB was of great clinical value, however only limited studies focused on this issue in previous literatures. Therefore, the present study was aimed to investigate the value of serial CL measurement in prediction of SPTB in women without short mid-trimester cervix. This study was a secondary analysis of open data from a previous research, which was the largest research in literature on serial mid-trimester CL measurement in post-conization pregnancy^14^.

A total of eight parameters derived from serial CL measurement in the mid-trimester were analyzed in our study, including CL_A_, CL_B_, CL_C_, CL_MAX_, CL_MIN_, %ΔCL_AB_, %ΔCL_BC_, and %ΔCL_AC_. After univariate and multivariate analysis, only CL_MAX_ and %ΔCL_AC_ were proven to be independent variables in predicting subsequent SPTB. Lower CL_MAX_ (OR [95%CI]: 0.92 [0.90–0.93]) and higher %ΔCL_AC_ (OR [95% CI]: 1.05 [1.01–1.09]) were related to an increasing risk of SPTB.

For parameters as CL measurement at single timepoint, CL_C_, CL_MIN_ and CL_MAX_ were significantly different between SPTB group and term birth group. Whereas only CL_MAX_ was selected as an independent variable in predicting subsequent SPTB on logistic regression analysis. To our knowledge, this finding is the first evidence in literature for the predictive value of CL_MAX_ in SPTB. Using ROC curve method, the AUC was 0.611 and the optimum cut-off value was <30 mm for CL_MAX_ (Sensitivity = 26.7%, Specificity = 90.8%, PPV = 18.8%, NPV = 94.0% and LR = 2.91).

In contrast, the value of CL_MIN_ has already been studied in previous researches. Owen *et al*. reported that CL_MIN_ in serial measurements significantly improved the prediction of SPTB compared with a single CL measurement^12^. However, our results indicated the superiority of CL_MAX_ to CL_MIN_ in predicting subsequent SPTB, which may be attributable to the exclusion of cases with short cervix <25 mm in our study. CL_MIN_ was a parameter aimed to improve the detection of short cervix in serial screening compared to single measurement in the mid-trimester. Therefore, the predictive value of CL_MIN_ may be dependent on the presence of short cervix <25 mm. Whereas CL_MAX_ was a parameter reflecting the upper limit, but not lower limit, of CL in mid-trimester, which may be independent on the presence of short cervix <25 mm. Thus, it seems to be reasonable that CL_MAX_ is of value in predicting subsequent SPTB in the subpopulation of pregnant women who did not have short mid-trimester cervix.

For parameters as CL change between two timepoints, %ΔCL_AC_ was an independent variable in predicting subsequent SPTB by univariate and multivariate analysis. Using ROC curve method, the AUC was 0.600 and the optimum cut-off value was ≥10% for %ΔCL_AC_ (Sensitivity = 42.9%, Specificity = 71.2%, PPV = 9.7%, NPV = 94.5% and LR = 1.49). This finding was consistent with previous studies^9,15–17^. Moroz *et al*. reported that the rate of change in CL was associated with the occurrence of subsequent SPTB^17^. For every 1 mm of cervical shortening between measurements, there was a 3% increase in odds of SPTB. However, in their research this association between change in CL and risk for SPTB was only present for women with short cervix <25 mm, but not for women with CL >25 mm. In contrast, in our study, %ΔCL_AC_ was still associated with an increasing risk of SPTB in the absence of short cervix (OR [95%CI]: 1.05 [1.01–1.09]). This difference may be attributable to the different duration of cervical screening in the mid-trimester. In the study of Moroz *et al*., CL measurements were performed between 24 weeks (range: 21–28 weeks) and 28 weeks (range: 25–33 weeks) of gestation. Whereas in our study, %ΔCL_AC_ represented the change of CL between timepoint A (range: 13–16 weeks) and timepoint C (range: 20–23 weeks). By including the early-stage of mid-trimester, the time interval between measurements in our study was much longer than that in Moroz *et al*.’s study (7 weeks vs 4 weeks on average interval). In support of this explanation, the change of CL during a short duration in our study, represented by %ΔCL_AB_ and %ΔCL_BC_, was also not associated with an increasing risk of SPTB.

Several limitations need to be acknowledged in our study. First, the study was a secondary analysis of a previous clinical research, which was subject to inherent limitations of the post hoc analysis. Secondly, another important limitation of the study was related to its retrospective design. Particularly in the earlier years of the study, when the association between conization and preterm birth was not well-established, preterm clinic referrals were unlikely to reflect all women post-conization within the population.

In conclusion, our study for the first time in literature reported the value of serial cervical length measurement in prediction of SPTB in post-conization pregnancy without short mid-trimester cervix. In the subpopulation of pregnant women who did not have short mid-trimester cervix, CL_MAX_ and %ΔCL_AC_ were of value in predicting SPTB, which warranted further investigations.

## Methods

### Study population

The study was a secondary analysis of the open data from a previous study, which was the largest research in literature on serial mid-trimester CL measurement in post-conization pregnancy^14,18^. The secondary analysis utilized de-identified patient information and was approved by the institutional review board of Peking Union Medical College Hospital.

The original database included a total of 725 pregnant women post-conization from the preterm surveillance clinics at three London University maternity units (Queen Charlottes Hospital, St Marys Hospital, Chelsea Westminster Hospital) between January 2004 and January 2014. Women were only eligible if they had their first singleton pregnancy after excisional cervical treatment for CIN of depth ≥12 mm (including Cone biopsy, LLETZ and LEEP). Any women with a prior preterm delivery (<37 weeks), mid-trimester miscarriage (>13 weeks), uterine anomaly or a multi-fetal pregnancy were excluded.

According to the aim of our study, women who had treatment for short mid-trimester cervix (n = 98) and short mid-trimester cervix without relevant treatment were excluded (n = 14). Finally, a total of 613 pregnant women post-conization were included in present study.

A pre-specified surveillance protocol including serial mid-trimester CL measurements was applied to all women attending prematurity clinics across sites for the duration of the study. CL measurements were taken at transvaginal ultrasound with an empty bladder, avoiding undue pressure on the cervix, and fundal pressure applied to illicit any further cervical shortening. The serial CL measurements were available for at least one of three screening timepoints; A: 13 + 0–15 + 6 weeks, B: 16 + 0–18 + 6 weeks, and C: 20 + 0–22 + 6 weeks.

### Statistical analysis

#### Predictor selection

CL measurements at different timepoints (CL_A_, CL_B_, CL_C_), the maximum and minimum CL measurements (CL_MAX_, CL_MIN_), and the percentage change in CL measurement between different timepoints (%ΔCL_AB_, %ΔCL_BC_, %ΔCL_AC_) were taken as the candidates for SPTB predictors.

First, in univariate analysis all above parameters were analyzed by Mann Whitney tests to identify the variables with a significant difference between SPTB group and term birth group. Subsequently, logistic regression with the backward elimination method for a multivariate analysis was performed to exclude the variables that were not independently significant for predicting the outcome. After the 2-step selection, the remaining predictors were used to assess the value of mid-trimester serial CL measurement in prediction of SPTB.

#### Diagnostic performance

For each above-selected predictor, the diagnostic test was used to calculate test characteristics such as sensitivity, specificity, PPV, NPV and LR from a 2 × 2 table. Finally, the ROC curve was plotted and its AUC was calculated.

All statistical analyses were performed using SPSS version 16.0. Two-tailed P values less than 0.05 were considered statistically significant.
